# Features of Structure and Flow Field in Homemade Co-Current Cavitation Water Jet Nozzle

**DOI:** 10.3390/ma18010146

**Published:** 2025-01-02

**Authors:** Chenhao Guo, Xing Dong, Haorong Song, Yun Jiang

**Affiliations:** 1 School of Safety Engineering, Heilongjiang University of Science and Technology, Harbin 150022, China; 2 School of Mechanical Engineering, Heilongjiang University of Science and Technology, Harbin 150022, China; dongxingwrh@163.com (X.D.); 13754623001@163.com (H.S.); 3 School of Mining Engineering, Heilongjiang University of Science and Technology, Harbin 150022, China; jiangyun011010@163.com

**Keywords:** co-current cavitation water jet nozzle, numerical simulation, pseudo-color image, cavitation cloud

## Abstract

The cavitation water jet cleaning and coating removal technique represents an innovative sustainable method for cleaning and removing coatings, with the nozzle serving as a crucial component of this technology. Developing an artificially submerged nozzle with a reliable structure and excellent cavitation performance is essential for enhancing cavitation water jets’ cleaning and coating removal efficacy in an atmosphere environment (non-submerged state). This study is based on the shear flow cavitation mechanism of an angular nozzle, the resonance principle of an organ pipe, and the jet pump principle. A dual-nozzle co-current cavitation water jet nozzle structure was designed and manufactured. The impact of the nozzle’s inlet pressure on the vapor volume percentage, as well as the axial and radial velocities inside the flow field, were examined utilizing ANSYS Fluent software with the CFD method. The dynamic change rule of the cavitation cloud is derived by analyzing the picture of the cavitation cloud in the nozzle’s outflow field utilizing pseudo-color imaging techniques. The results show that the maximum vapor volume percentage is more significant than 95% for different inlet pressures in the internal nozzle. The changes that occur in the cavitation cloud exhibit notable regularity, including the four stages of cavitation, which are inception, development, shedding, and collapse. A change period is 1.5 ms, which proves that the homemade co-current cavitation water jet nozzle can achieve good cavitation effects.

## 1. Introduction

Cavitation is a prevalent occurrence in fluid dynamics and was first identified as a significant impediment to the effective functioning of hydraulic machinery. It adversely impacts domains such as engineering and medicine [1,2,3]. As the research progressed, the researchers not only found the cause of cavitation but also ingeniously utilized the high-intensity energy generated by the collapse of cavities, developing it into an inexpensive, clean, and environmentally friendly manufacturing technology, cavitation water jet technology [4,5]. Cavitation water jet technology uses high-pressure water as a carrier. When high-pressure water flows through a specific nozzle configuration, the surrounding absolute pressure falls below the saturated vapor pressure of water, forming numerous cavitation bubbles inside the water. When cavitation bubbles strike a target surface by a jet, it has a dual effect, namely the pressure pulsation from the collapse of the cavitation bubble and the erosive damage caused by the high-velocity jet. It has higher impact force, destructive power, and lower energy consumption than ordinary water jets. Consequently, it has been extensively utilized in areas such as effective cleaning and coating removal, shot peening, and material cutting [6,7,8,9,10].

The critical component of the water jet that generates cavitation is the nozzle, and the nozzle’s structural design directly affects the jet’s cavitation effect and cleaning and coating removal performance. According to the different principles of cavitation priming, the conventional submerged cavitation nozzle is divided into shear-type, bypassing-type, and oscillating-type nozzles [11]. Researchers can promote cavitation’s initial formation and development by selecting a reasonable nozzle structure and adjusting the jet’s target distance. Energy is released when the cavitation bubbles collapse at the jet’s impact target, thus achieving the effect of cavitation damage [12]. Soyama H [13] conducted cavitation erosion tests using eight cavitation nozzles with different structures. The erosion rate of the target per unit of time was used as the evaluation index for examining the impact of various nozzle geometries upon the intensity of the cavitation jet. The test results showed that the submerged cavitation nozzle, throat diameter *d*, exit diameter *D*, and length *l* affect cavitation erosion intensity. When *d*:*D*:*l* = 1:8:8, the nozzle’s cavitation intensity is maximal, proving that the nozzle’s geometry significantly affects the cavitating jet’s intensity. Panda et al. [14] designed a new submerged cavitation nozzle with an outlet diffusion to improve the jet’s cavitation effect without increasing energy consumption. Comparative numerical simulations indicate that the new type of submerged cavitation nozzle with an outlet diffusion section generates three times the cavitation intensity of a regular cylindrical nozzle and 1.5 times the intensity of a venturi nozzle, proving that this structure can significantly enhance the jet cavitation effect. Dong et al. [15] employed CFD fluid dynamics software to numerically simulate an ordinary submerged cavitation nozzle, investigating the effects of the nozzle structure on the vapor volume percentage. The simulation findings demonstrate that when the input pressure is 20 MPa, the nozzle contraction angle *α* is 13.5°, and the expansion angle *β* is 60°, the vapor volume percentage from the nozzle can attain 95%. The cavitation cloud forms adjacent to the edge of the diffusion segment at the nozzle’s output and disperses in the configuration of a vortex ring.

As the current angular cavitation nozzle, the swirling cavitation nozzle, and the self-oscillating pulse cavitation nozzle structures can only obtain good cavitation cleaning results under certain submerged conditions [16], under non-submerged conditions, the cavitation generated by the above nozzles mainly exists inside the nozzle. The cavitation effect rapidly diminishes as the jet exits the nozzle and enters the atmosphere. The energy produced during the collapse of the cavitation bubble is not entirely harnessed, leading to a limited effective target range of the jet. Furthermore, some large and critical parts cannot be immersed in water for cavitation jet treatment, thereby restricting the application of the cavitation jet in industrial production [17]. To solve this problem, researchers have successively proposed a variety of co-current artificial submersion cavitation nozzle structures, aiming to overcome the limitations of the submerged condition of the cavitation jet, increase the effective target range, and increase the applicability of the cavitation jet in industrial production. Soyama [18] designed a co-current overlapping nozzle structure, which allows shear cavitation to form at the annular interface where the internal nozzle’s high-pressure water converges with the external nozzle’s low-pressure water. The low-speed water jet replaces the static water in the traditional submerged jet, and this new type of artificial submerged nozzle initially achieves a cavitating jet without the need for submergence. Marcon et al. [19] obtained the cavitation performance and shot peening effect of a co-flow cavitation water jet nozzle via a cavitation jet striking force test, revealing that the maximum residual stress generated by the co-flow cavitation water jet nozzle striking on the 7075-t651 aluminum alloy target can reach 401 MPa. Vidvans et al. [20], to improve the cavitation performance of a co-current cavitation water jet nozzle, designed a co-current cavitation water jet nozzle structure with an organ pipe-type inner nozzle and characterized its cavitation strength by the bubble cloud visualization test and the residual stress of the target. The findings demonstrate that the cavitation strength of the nozzle exceeds that of the conventional cavitation nozzle by 61%, and the residual compressive stress is more significant than that of the conventional cavitation nozzle, which proves that the co-current cavitation water jet nozzle can achieve a better cavitation effect under the non-submerged condition. Wu et al. [21] investigated the viability of an artificially immersed cavitation jet for enhancing the 7075 aluminum alloy by examining the surface appearance and roughness of the target material at varying jet blasting velocities to ascertain the effects of the new jet blasting on its microstructure. The findings reveal that the shearing impact of high- and low-velocity jets can produce a bubble cloud when a cavitating jet is artificially immersed. It can overcome the conventional submerged cavitation jet target distance of insufficient defects and effectively impact the target surface plastic deformation to increase its strength for the application of artificial submerged cavitation jet target surface strengthening to provide technical support. Luo et al. [22] used ANSYS Fluent to numerically simulate the flow field of a co-current double-nozzle artificial submerged cavitation nozzle in order to investigate the cavitation shot peening ability of an artificial submerged nozzle. The influence of the internal nozzle geometry and inlet pressure on the vapor volume fraction was obtained. It was found that the inlet pressure was positively correlated with the vapor volume fraction. The convergence angle of the internal nozzle was negatively correlated with the vapor volume fraction, providing a reference for the structural design of the artificial submerged nozzle. Fan et al. [23] designed a double-current cavitation water jet nozzle structure to improve the cavitation jet’s rock-breaking capability. The internal nozzle is a cavitation nozzle that can be filled with abrasive, and the external nozzle is a co-current low-speed jet nozzle structure. High-speed camera technology and target erosion experiments were used to investigate the rock-breaking performance of the nozzle under different jet pressures, and the effects of rock-breaking time and nozzle diameter on the cavitation performance were discussed. The results show that when the inlet pressure of the internal high-pressure cavitation nozzle is 50 MPa, the inlet pressure of the external co-current low-speed jet nozzle is 0.18 MPa; the low-speed and high-speed jets have the best effect of shear cavitation, which can erode rock bodies up to a depth of 600 μm. It provides a reference for applying co-current cavitation water jet nozzles in the field of rock breaking.

An artificially submerged cavitation jet nozzle structure comprises two inlet channels, one for high pressure and the other for low pressure; the flow structure is very complex and the jet is in the form of intense turbulence [24,25], which involves complex interphase interactions (cavitation bubble–cavitation bubble, cavitation bubble–liquid phase), so conventional experimental methods are challenging to capture the details in the vapor bubble motion and characterize the dynamic change pattern of the vapor cloud. Due to the swift advancement of computer technology and the emergence of high-resolution image display instruments, researchers have used computational fluid dynamics (CFD) software and high-speed camera technology for numerical simulation and real-time acquisition of the cavitation flow field, respectively, to realize the calculation and visualization of the flow characteristics of the cavitation jet flow field [26,27]. Sekyi et al. [28] analyzed the variation rule of the cavitation nozzle’s bubble cloud based on CFD and high-speed camera technology and established the kinetic mathematical model of individual cavitation bubbles, and the comparison study of the grayscale pictures processed in MATLAB indicates that an increase in nozzle diameter and low pressure negatively influences the eroding action of the cavitating jet on the target. Du et al. [29] designed an artificially submerged cavitation nozzle based on the angular nozzle structural design principle. They performed CFD numerical simulations, which showed that the high-pressure water jet produces a significant cavitation effect at the jet boundary in the artificially created submerged environment, which can be maintained in the atmosphere. Li et al. [30] employed CFD techniques and impact tests to examine the effects of the self-made artificially immersed cavitating nozzle’s geometrical factors, target distance, and inlet pressure on cavitation intensity. The data indicate that the shear effect formed between high-velocity and low-velocity is the core factor in producing cavitation incipient. The optimal cavitation capability of this nozzle occurs when the convergence angles *α* of the interior nozzle and *β* of the exterior nozzle are both 30°, with a target distance of 30 mm. Zheng [31] designed a two-stage series-connected double-chamber organ pipe cavitation nozzle structure. The cavitation capacity of the nozzle under different inlet pressures, diffusion angles, and diffusion part lengths was obtained using the CFD method. The outcomes indicate that the nozzle’s diffusion angle exerts a more pronounced influence on cavitation capacity, velocity magnitude in the jet reflux region, and length of the isocore compared to inlet pressure and the length of the diffusion part. Onish et al. [32] integrated a CFD numerical simulation with rapid digital imaging to examine the cyclic dynamics of cavitation cloud creation, growth, and shedding produced by a cavitation nozzle. The results show that a pair of annular clouds composed of initial and subsequent newborn cavitation clouds are shed downstream in sequence. This process is cyclic reciprocity, and the Strouhal number, which dominates the shedding of the cavitation cloud, is stable at 0.21–0.29, which validates the precision of the computer-simulated outcomes. It has a particular value of reference for the improvement of the structural performance of the cavitation jet nozzle.

In summary, while certain researchers in this domain have conducted structural optimization, computational simulations, and rapid speed photography tests on artificially immersed cavitation nozzles, current research has not yet explored the structure of an artificially immersed cavitation nozzle featuring an organ pipe self-oscillating angular cavitation nozzle for the inner nozzle and conical–cylindrical combination nozzles for the outer nozzle. This study aims to eliminate the submerged condition limitation under the conventional cavitation water jet working condition and enhance the nozzle’s cavitation capability. Based on the shear flow cavitation mechanism of the angular nozzle, the resonance principle of the organ pipe, and the principle of the jet pump, a new type of co-current cavitation water jet nozzle is designed. A numerical simulation of the nozzle’s flow field is conducted using the CFD method, revealing the influence of inlet pressure on the vapor volume percentage, axial velocity, and radial velocity of the flow field. A comparative examination with the existing co-current cavitation nozzle structure to validate the efficacy of the homemade co-current cavitation water jet nozzle structure is made. This is with the aid of high-speed photographic equipment to capture the morphology and distribution of the artificial submerged cavitation jet cloud, thereby enhancing technical assistance for the advancement of theory and implementation of the artificial submerged cavitation water jet.

## 2. Materials and Methods

### 2.1. Parameters for Simulation Configuration

A numerical simulation of the water–water vapor two-phase flow field of a homemade co-current cavitation nozzle was carried out by using the CFD method and ANSYS Fluent 2022 R2 software. In the CFD computer simulation, water was chosen as the primary phase, with a density of 1000 kg/m^3^ and a viscosity of 1 × 10^−3^ Pa·s, and water vapor was chosen as the secondary phase, with a density of 2.558 × 10^−2^ kg/m^3^ and a viscosity of 1.26 × 10^−5^ Pa·s. The impact of pressure on the properties of the jet cavitation flow field was examined by varying the inlet pressure of the homemade co-current cavitation nozzle.

### 2.2. Structural Design of Co-Current Cavitation Water Jet Nozzle

#### 2.2.1. Overall Structure

**Figure 1:**
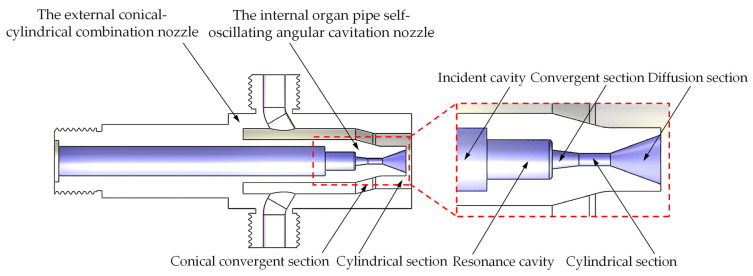
Schematic structure of homemade co-current cavitation water jet nozzle.

**Figure 2:**
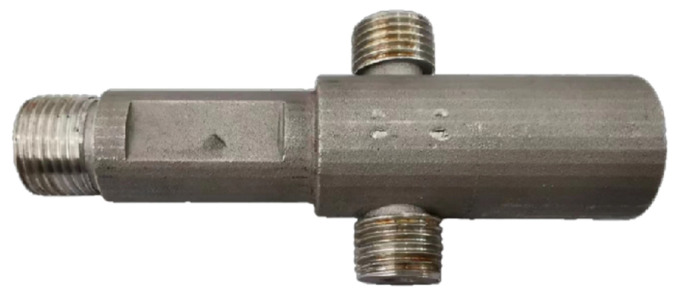
Homemade co-current cavitation water jet nozzle physical picture.

The homemade co-current cavitation water jet nozzle structure is schematically shown in Figure 1. As seen in Figure 1, the overall nozzle structure consists of two parts, the internal organ pipe self-oscillating angular cavitation nozzle and the external conical–cylindrical combination nozzle structure. The internal organ pipe self-oscillating angular cavitation nozzle is designed based on the angular nozzle shear flow cavitation mechanism and the organ pipe resonance principle and consists of an incident cavity, resonance cavity, convergent section, cylindrical section, and diffusion section; the external nozzle structure comprises a conical convergent portion and a cylindrical segment. The physical photo of the homemade co-current cavitation water jet nozzle structure is shown in Figure 2.

This study of a homemade combined co-current cavitation water jet nozzle has two advantages. Firstly, the internal organ pipe self-oscillation angular cavitation nozzle with the help of the organ pipe spray group resonance cavity of the feedback mechanism and amplification mechanism of the high-pressure water pulsation double modulation results in high-frequency pressure oscillation, which leads to the generation of cavitation. At the same time, the use of the angular cavitation nozzle outlet diffusion section shear effect of the nozzle outlet flow field to produce many vortices further enhances the role of the cavitation composite. Secondly, the conical convergent section of the external conical–cylindrical combined nozzle aids in the uniform introduction of the low-pressure jet, ensuring the wrapping effect of the peripheral flow beam on the internal high-speed jet. The cylindrical section structure can guide the external flow beam, promote the development of cavitation bubbles, ensure jet convergence, and significantly contribute to the mitigation of the reduction in axial velocity.

#### 2.2.2. Structural Design of Internal Organ Pipe Self-Oscillating Angular Cavitation Nozzle

**Figure 3:**
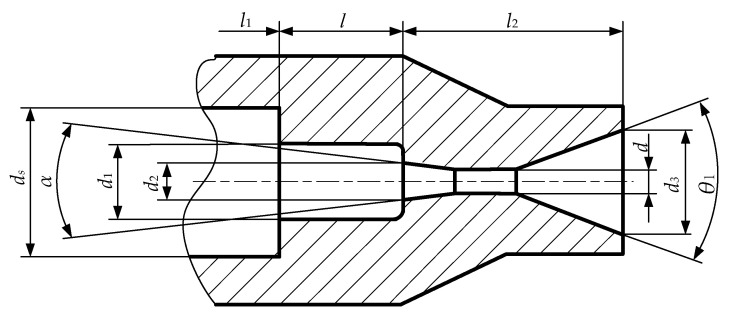
Dimensions of the internal organ pipe self-oscillating angular cavitation nozzle.

Figure 3 gives the internal organ pipe self-oscillating angular cavitation nozzle dimensions; the internal organ pipe self-oscillating angular cavitation nozzle is based on the angular nozzle shear flow cavitation mechanism and the organ pipe resonance principle, the traditional resonance cavity outlet cavity cylindrical contraction section is changed to a conical convergence section of the transition, and the angle of convergence α to choose the best recognized is 13.5° [23]. Only when the nozzle outlet cylindrical segment’s diameter meets certain conditions can the pressure within the nozzle’s flow field drop below the saturated vapor pressure, resulting in cavitation. Given a predetermined inlet pressure and flow rate, the nozzle’s outlet diameter *d* can be as follows [33]:

(1) $$d=\sqrt{\frac{4Q}{\sqrt{\frac{2p}{\rho }}\pi \mu }}$$

where *Q* denotes the internal organ pipe self-oscillating angular cavitation nozzle’s flow rate (m^3^/s); *μ* denotes the fluid flow factor; *p* denotes the internal organ pipe self-oscillating angular cavitation nozzle’s inlet pressure (Pa); and *ρ* denotes the water’s density (kg/m^3^).

Substituting the actual working condition parameters, the nozzle’s outlet diameter *d* can be obtained as 1.6 mm. According to the internationally commonly used angular cavitation water jet nozzle design in the proportional relationship between the nozzle diameter *d* and the length *l*_2_, i.e., *d*:*l*_2_ = 1:8 [13], then the internal organ pipe self-oscillation angular cavitation nozzle angular nozzle section length *l*_2_ is taken as 12.8 mm.

The role of the resonant cavity of the internal cavitation nozzle in this study is to control the pressure gradient between the nozzle inlet cavity section and the outlet constriction section, to delay the position of cavitation occurrence, and to avert a substantial quantity of cavitation bubbles from obstructing the fluid flow pathway as a result of premature cavitation. On the other hand, the resonant cavity can match its intrinsic frequency and the critical self-excited structural frequency of the fluid to generate a self-oscillation phenomenon. The resonant cavity structure is a self-excited oscillatory amplifier consisting of length *l* and diameter *d*_1_, where the inlet contraction section (*d*_s_/*d*_1_)^2^ and outlet contraction section (*d*_1_/*d*_2_)^2^ are the key parameters determining the cavitation effect of self-excited oscillation.

The internal organ pipe self-oscillating angular cavitation nozzle resonant cavity intrinsic frequency *f* is determined by the following equation [34]:

(2) $$f=K_{N}\frac{a}{l}$$

where *a* denotes the altered wave speed within the resonant cavity of the organ pipe (m/s); *l* denotes the cavity length of the organ pipe-type resonant cavity (mm); and *K_N_* denotes the modulus coefficient.

The modulus factor *K_N_* is calculated as [35]

(3) $$K_{N}=F\left\{N,{\left(\frac{d_{s}}{d_{1}}\right)}^{2},{\left(\frac{d_{1}}{d_{2}}\right)}^{2}\right\}=\left\{\begin{array}{cc} \frac{2N-1}{4} & \left[{\left(\frac{d_{s}}{d_{1}}\right)}^{2}\geq 1,{\left(\frac{d_{1}}{d_{2}}\right)}^{2}\geq 1\right]\\ \frac{N}{2} & \left[{\left(\frac{d_{s}}{d_{1}}\right)}^{2}\geq 1,{\left(\frac{d_{1}}{d_{2}}\right)}^{2}\geq 1\right] \end{array}\right\}$$

where *N* denotes the coefficient of oscillation within the resonant cavity, *N* = 1; *c* denotes the value determined by the experiment; *d*_s_ denotes the incident cavity cross-section’s diameter (mm); *d*_1_ denotes the resonant cavity cross-section’s diameter (mm); and *d*_2_ denotes the conical convergence section at the exit of the resonant cavity’s diameter (mm).

The critical self-excited structure frequency f* of a self-oscillating cavitating jet is expressed in terms of the critical Strouhal number Sr* of the nozzle as [34]

(4) $$Sr^{*}=f^{*}\frac{d_{2}}{v}$$

where *v* denotes the nozzle outlet jet velocity (m/s).

Normally the Mach number *Ma* is expressed as [34]

(5) $$Ma=\frac{v}{a}$$

Associative Equations (4) and (5) lead to

(6) $$f^{*}=Sr^{*}\frac{Ma}{d_{2}}a$$

The organ pipe will generate the most robust resonance when the intrinsic frequency *f* of the resonant cavity of the internal organ pipe self-oscillating angular cavitation nozzle and the critical self-excited structure frequency f* of the jet are equal. The joint Equations (2) and (6) can be obtained as follows:

(7) $$\frac{l}{d_{2}}\approx \frac{K_{N}}{MaSr^{*}}$$

According to Crow et al. [36], the results of the jet test show that the conditions that can make the oscillating cavity produce strong resonance are a Mach number *Ma* in the range of 0.08 to 0.10 and when the critical Strouhal number Sr* is equal to 0.3 or an integer multiple of 0.3 [34]. At this time, the nozzle cavitation effect is optimal. Therefore, in this study, when the Mach number *Ma* is taken as 0.08, the critical Strouhal number Sr* is taken as 0.9, and the resonant cavity exit cone convergence section’s diameter *d*_2_ is taken as 2.4 mm, the resonant cavity’s length is calculated to be *l* = 8.3 mm.

In this study, considering the high-pressure piping dimensions of the existing test-bed jet system, to achieve a better installation connection and flow stabilization, the internal organ pipe self-oscillating angular cavitation nozzle is taken to have an inlet cavity diameter *d_s_* of 10 mm, and the inlet cavity length *l*_1_ is extended to be 70 mm; according to (*d*_s_/*d*_1_)^2^ ≥ 1, (*d*_1_/*d*_2_)^2^ ≥ 1 and the optimal cross-section shrinkage ratio reasonable value range of 3.5–4.5 of the design criterion [37], the resonant cavity inlet diameter *d*_1_ is calculated to be 5 mm; the expansion angle *θ*_1_ is taken to be 40° considering the jet cavity morphology, axial length, and drag coefficient.

#### 2.2.3. Structural Design of External Conical–Cylindrical Combination Nozzle

The external conical–cylindrical combination nozzle is manufactured according to the jet pump concept and the co-current overlapping cavitation nozzle design principle of artificial submergence. Through experimental studies, researchers found that the structural dimensions of the external nozzle of the artificially submerged cavitation nozzle have a crucial influence on the cavitation jet effect produced [38], so key parameters such as the axial distance *H* between the inner and outer nozzles, the outlet diameter *D*_0_ of the outer nozzle, and the conical convergence angle *θ*_2_ are defined by the structural parameters of the internal organ pipe self-oscillating angular cavitation nozzle.

The formula for calculating the outlet diameter *D*_0_ of external conical–cylindrical combination nozzles [39,40] is as follows:

(8) $$D_{0}=\sqrt{\delta m_{y}}d_{3}$$

where *δ* denotes the coefficient of nozzle jet area contraction, taking the value of 0.62; *m_y_* denotes the ratio of inner and outer nozzle exit area, taking the value of 9.1; and *d*_3_ denotes the internal nozzle outlet’s diffusion section’s cross-sectional diameter (mm).

The outer nozzle’s optimal outlet length, *L_kopt_*, was studied experimentally with different area ratios and operating pressures [39].

(9) $$L_{kopt}=1.02\sqrt{\delta m_{y}}d_{3}$$

The conical convergence angle *θ*_2_ of the external conical–cylindrical combination nozzle is taken as 30°, and the dimensions of the axial spacing *H* between the internal and external nozzle exits are taken as 1.5 mm. To reduce energy loss and disturbance during the introduction of low-pressure water flow, the double diversion port adopts an axisymmetric slant design. The angle *θ*_3_ between the double diversion port and the axis line is 60°, and the diversion port’s diameter is *d_y_* = 6 mm.

### 2.3. Method of Computation and Establishment of Boundary Conditions

Given that the configuration of the homemade co-current cavitating water jet nozzle features co-current high- and low-pressure water ingress and acknowledging the complexity of the cavitating water jet as an unsteady flow, a high-efficiency pressure solver is employed. In the discrete solution of the specific pressure term equation, the PRESTO! format is selected. For the discrete solution of the volume fraction, the quick format is selected, and the second-order upwind format is used for all other equations; the convergence criterion is set as the residuals *R* ≤ 10^−6^ and the relaxation factor is adopted as the default. The coupled method of pressure and velocity is adopted as the coupled algorithm [41].

The high-pressure water inlet of the internal organ pipe self-oscillating angular cavitation nozzle and the two low-pressure water inlets of the external conical–cylindrical combination nozzle are set as pressure inlets, and the boundary of the flow field outside the outlet of the homemade co-current cavitation water jet nozzle is set as a pressure outlet.

## 3. Mathematical Pattern for Simulation Computations

### 3.1. Multiphase Flow Model

The internal flow field of the homemade co-current cavitating nozzle is a biphasic flow of water and water vapor. The water and water vapor phases of the medium penetrate and interact with each other. The two-phase medium is approximated to be a continuous medium. A large amount of vortex cavitation bubbles is discretely distributed, and the volume ratio of cavitation bubbles is less than 10% in the whole flow field. This study utilizes the mixture model as a multiphase flow model to simulate the complete flow field of the homemade co-current cavitating nozzle. Moreover, the cavitation model in the mixture model is chosen to perform the interphase mass conversion and make the numerical simulation results closer to reality. A cavitation water jet is a form of fluid motion that must satisfy the continuity, momentum, and second-phase volume fraction equation [42].

(1) The continuity equation for the water and water vapor phases is
  (10) $$\frac{\partial \rho_{m}}{\partial t}+\nabla \cdot (\rho_{m}v_{m})=0$$
  (11) $$\rho_{m}=\sum_{k=1}^{2}\alpha_{a}\rho_{a}$$
  where *ρ_m_* denotes the hybrid-phase density (kg/m^3^); *v*_m_ denotes the hybrid-phase velocity (m/s); *t* denotes time (s); α*_a_* denotes the volume fraction of the water vapor phase; and *ρ_a_* denotes the density of the water vapor phase (kg/m^3^).
(2) The momentum equation for the water and water vapor phases is
  (12) $$\frac{\partial \left(\rho_{m}v_{m}\right)}{\partial t}+\nabla \cdot \left(\rho_{m}v_{m}v_{m}\right)=-\nabla p+\nabla \cdot \left[\mu_{m}\left(\nabla v_{m}+\nabla {v_{m}}^{T}\right)\right]+\rho_{m}\vec{g}+F+\nabla \cdot \left(\sum_{k=1}^{2}\alpha_{a}\rho_{a}v_{dr,a}v_{dr,a}\right)$$
  where *p* denotes the operational pressure (Pa); *μ_m_* denotes the hybrid-phase viscosity (Pa·s); g denotes the acceleration of gravity (m/s^2^); *F* denotes the volume force (Pa/m^3^); and *v_dr,a_* denotes the water vapor phase’s escape velocity (m/s).
(3) The water vapor volume percentage equation is
  (13) $$\frac{\partial \left(\alpha_{a}\rho_{a}\right)}{\partial t}+\nabla \cdot \left(\alpha_{a}\rho_{a}v_{m}\right)=-\nabla \cdot \left(\alpha_{a}\rho_{a}v_{dr,a}\right)+\sum_{a=1}^{n}m_{aw}-m_{wa}$$
  where *m_aw_* denotes the molecular interaction force from the water vapor phase to the water phase and *m_wa_* denotes the molecular interaction force from the water phase to the water vapor phase.

### 3.2. Turbulence Model

The turbulence model directly affects the jet flow characteristics and the cavitation bubble morphology. Considering the substantial velocity disparity among the inside and outside flow fields of a co-current cavitation water jet nozzle, which results in the formation of a jet shear layer characterized by a high Reynolds number in both the inside and outside flow bundles, the RNG *k*-*ε* model, which is more accurate and adaptive in calculating the cavitation vortex structure, was adopted in the present study to solve for simulation [43].

(1) Based on the equation, the kinetic energy of turbulent flow *k* gives
  (14) $$\frac{\partial \left(\rho_{m}k\right)}{\partial t}+\frac{\partial \left(\rho_{m}ku_{i}\right)}{\partial x_{i}}=\frac{\partial }{\partial x_{j}}\left[\alpha_{k}\left(\mu_{m}+\mu_{t}\right)\frac{\partial k}{\partial x_{j}}\right]+G_{k}+\rho_{m}\varepsilon$$
  where *μ_t_* denotes the turbulent viscosity (Pa·s); *G_k_* denotes the turbulent kinetic energy produced by the average velocity difference (m^2^/s^2^); and *ε* denotes the amount of loss of turbulent kinetic energy per unit of fluid (m^2^/s^3^).
(2) The equation for the dissipation rate *ε* is
  (15) $$\frac{\partial \left(\rho_{m}\varepsilon \right)}{\partial t}+\frac{\partial \left(\rho_{m}\varepsilon u_{i}\right)}{\partial x_{i}}=\frac{\partial }{\partial x_{j}}\left[\alpha_{\varepsilon }\left(\mu_{m}+\mu_{t}\right)\frac{\partial \varepsilon }{\partial x_{j}}\right]+C_{1\varepsilon }\frac{\varepsilon }{k}G_{k}-C_{2\varepsilon }\rho_{m}\frac{\varepsilon^{2}}{k}$$
  where *C*_1_*_ε_* and *C*_2_*_ε_* denote the experimentally measured constant coefficients.

### 3.3. Cavitation Calculation Model

The Zwart–Gerber–Belamri cavitation model accurately represents the cavitation bubble shape in a highly turbulent cavitation flow environment, offering excellent computational stability and quick resolution. Consequently, this model is employed in this work for computational modeling of the homemade co-current cavitation water jet nozzle’s flow field.

The model equation is [44]

When *P* ≤ *P_v_*,

(16) $$R_{e}=F_{vap}\frac{3\alpha_{nuc}\left(1-\alpha_{v}\right)\rho_{v}}{R_{B}}\sqrt{\frac{2}{3}\frac{\left(P_{v}-P\right)}{\rho_{1}}}$$

When *P* > *P_v_*,

(17) $$R_{c}{=F}_{\mathrm{cond}}\frac{3\alpha_{v}\rho_{v}}{R_{\mathrm{nuc}}}\sqrt{\frac{2}{3}\frac{\left(P-P_{v}\right)}{\rho_{1}}}$$

where *P* denotes the actual working condition surrounding pressure (Pa); *P_v_* denotes the saturated pressure within the cavitation bubble (Pa); *R_e_* and *R_c_* denote the rate of water vapor phase creation and condensation; *F_vap_* denotes the evaporation phase coefficient; *F_cond_* denotes the condensation phase coefficient; *α_nuc_* denotes the gas nucleus’s volume fraction; *R_B_* denotes the radius of the cavitation bubble (mm); and *ρ*_1_ denotes the water phase’s density (kg/m^3^).

## 4. Simulation Results and Discussion

### 4.1. Finite Element Model

**Figure 4:**
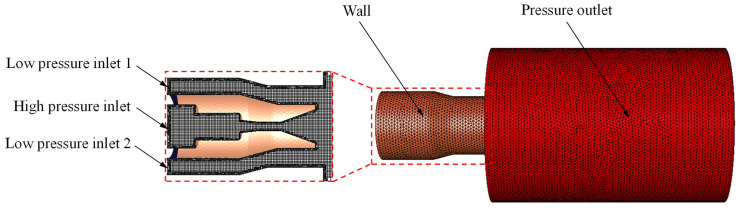
Finite element model.

This study utilized Solidworks 2020 software to build a three-dimensional representation of the interior and exterior flow fields of the co-current cavitation water jet nozzle. The Fluent Meshing program was utilized to execute the tetra unstructured meshing for the nozzle’s geometric model. The specific division method was as follows: firstly, Hexcore Octree tetrahedral unstructured mesh was used to divide the overall geometric model and secondly, BOI local mesh encryption was carried out for the nozzle outlet cylindrical section, as well as for the areas with complex flow characteristics, such as the intersection area of the inner and outer nozzles in the external flow field, to accurately capture the purpose of the small-scale vortices and air bubbles. Figure 4 illustrates the finite element model.

### 4.2. Mesh Irrelevance Test

The level of detail of the mesh is essential for the precision of numerical simulation calculations; therefore, in practical fluid dynamics calculation applications, by properly encrypting the mesh, the numerical simulation solution will gradually converge to the final accurate solution. However, the increasing number of meshes will seriously consume computer resources, corresponding to the rising time cost. Therefore, the finite element model needs a mesh irrelevance test to improve computational efficiency while maintaining accuracy. This study investigates the highest vapor volume percentage inside the flow field for five different mesh numbers with an external nozzle input pressure of 3 MPa and an internal cavitation nozzle input pressure of 10 MPa for the co-current cavitation water jet nozzle, and the outcomes are demonstrated in Table 1 after the computation reaches the steady state.

**Figure 5:**
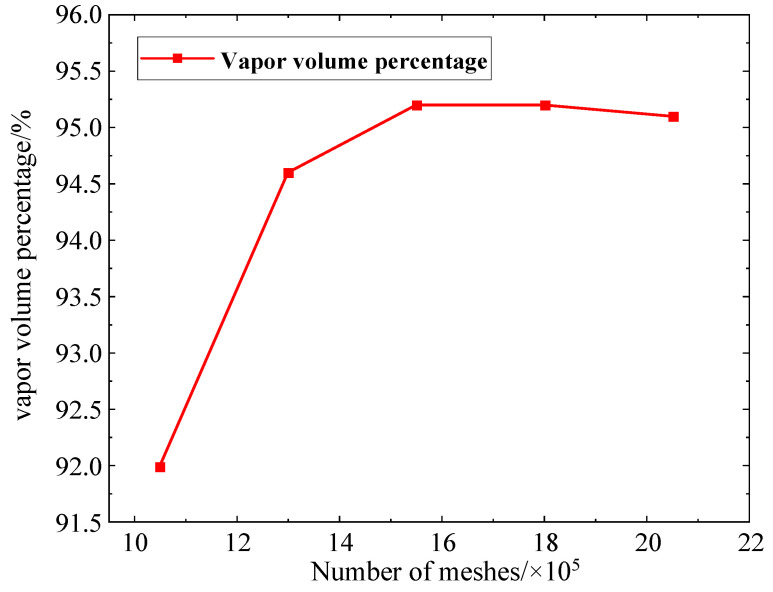
Variation curve of maximum vapor volume percentage for different numbers of meshes.

Figure 5 illustrates the variation curves of the maximal vapor volume percentage within the nozzle flow field for different mesh densities. According to the indication in Figure 5, when the quantity of meshes is fewer than 1,549,513, there is a notable disparity existing in the calculated maximal vapor volume percentage within the nozzle flow field; when the quantity of meshes is higher than or equivalent to 1,549,513, the continuing increase in the number of meshes on the calculation of the vapor volume percentage of the results of the difference is negligible. Considering the computational efficiency and accuracy of numerical simulation, the number of mesh cells of the nozzle model is determined to be 1,549,513.

### 4.3. Effect of Internal Nozzle Inlet Pressure on Maximum Vapor Volume Percentage

**Figure 6:**
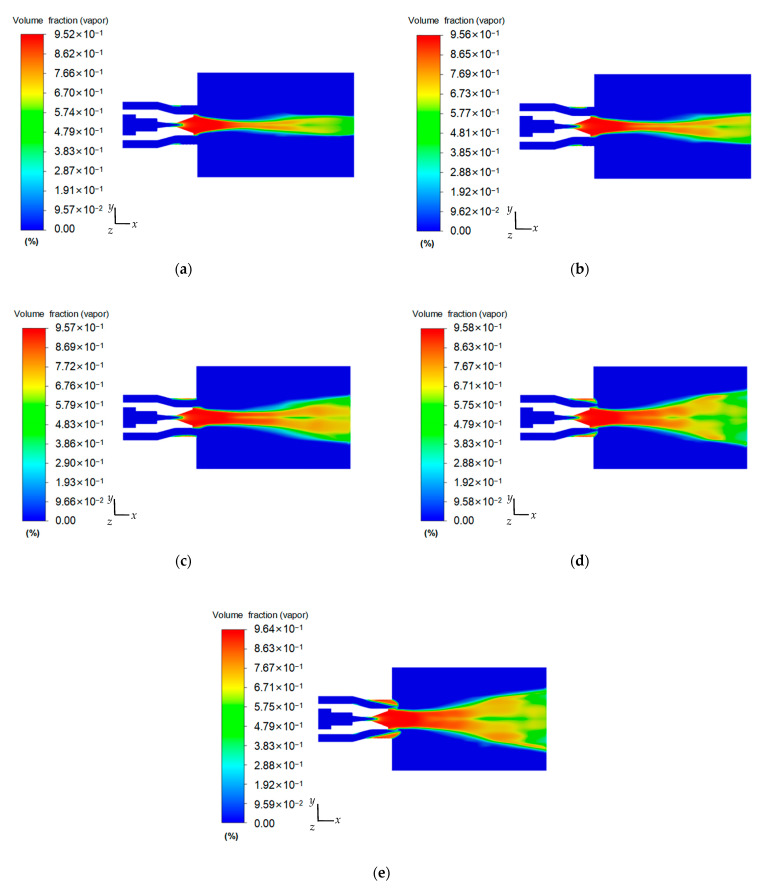
Cloud diagram of maximum vapor volume percentage distributions at (**a**) 10 MPa, (**b**) 15 MPa, (**c**) 20 MPa, (**d**) 25 MPa, and (**e**) 30 MPa.

The pressure at the input of the inner nozzle of the homemade co-current cavitation water jet nozzle provides energy for the creation of jet cavitation incipient conditions, and when the inlet pressures of the two low-pressure inlets of the external conical–cylindrical combination nozzle are kept constant, the vapor volume percentage within the overall flow farm and the jet velocity vary with alterations in the input pressure of the internal cavitation nozzle. Figure 6 illustrates the distribution of the highest vapor volume percentage inside the flow farm of the co-current cavitating water jet nozzle at an external nozzle input pressure of 3 MPa and internal nozzle input pressures of 10, 15, 20, 25, and 30 MPa.

As demonstrated in Figure 6, the vapor volume of the homemade co-current cavitation water jet nozzle has an axisymmetric distribution, and the vapor volume percentage gradually rises with the augmentation of the input pressure of the internal cavitating nozzle. Specifically, when water jets via the cylindrical section of the internal cavitation nozzle and enter the position of the wall of the outlet diffusion section, cavitation occurs due to the pressure gradient between the proximal wall portion of the outlet diffusion section and the central jet. When high-pressure water is expelled via the internal nozzle output to create a cavitating jet, the external low-pressure jet wraps the internal high-speed cavitating jet and enters the atmosphere. Owing to the significant velocity differential among the interior and exterior jets, a violent exchange of shear momentum occurs between the jets, causing the fluid within the jet shear region to form a vortex structure under the action of viscous forces and reverse pressure differences. The pressure at the center of the vortex continuously decreases. When the pressure decreases below the saturated vapor pressure of the environment, cavitation will occur and bubbles will be generated, thus promoting the cavitation effect. The tiny bubbles generated in the boundary layer will grow and move downstream with the jet. During the development of the movement, the bubbles will continuously combine with the primary bubbles to achieve a good jet cavitation effect under non-submerged conditions. This is consistent with the findings of Lei [27] and others that artificial submerged nozzle cavitation initially occurs at the exit cylindrical section and diffusion section, and the leading cause of cavitation is the shear interaction between the internal fast jet and the peripheral flow.

Figure 6 also illustrates the cavitation effect of the co-current cavitation water jet nozzle, which exhibits an upward trend with the input pressure of the internal nozzle. The greater the input pressure, the greater the pressure and velocity gradients among the interior high-pressure jet with the exterior low-pressure jet, the more significant the cavitation effect, and the greater the vapor volume percentage. When the input pressures are 10, 15, 20, 25, and 30 MPa, the maximal vapor volume percentages are 95.2%, 95.6%, 95.7%, 95.8%, and 96.4%, respectively, which proves that the homemade co-current cavitation water jet nozzle can produce a good cavitation effect. It is pertinent to note that when the inlet pressure of the internal nozzle rises, the momentum exchange among the interior high-pressure jet with the exterior low-pressure jet in the shear boundary layer becomes more intense, causing the length and range of the cavitation cloud in the flow field to expand. The size of the cavitation cloud penetrates the entire external flow field, proving that the cavitation bubbles produced by the homemade co-current cavitation nozzle can be effectively maintained in the atmosphere, eliminating the application limitation that the cavitation jet can only obtain better results in a submerged environment.

Figure 6 also demonstrates that the jet undergoes a significant energy exchange with the atmosphere during its downstream movement, gradually destabilizing its coherence. Due to the movement of the jet and changes in ambient pressure, the radial dimensions of the jet’s cavitation region decrease at a specific downstream position. As the jet continues to move downstream for a certain distance, the diffusion of the internal and outer jet bodies becomes more and more pronounced and the cavitation bubble group collapses over a wide area. When the input pressure of the internal nozzle exceeds or equals 20 MPa, a distinct cloud shedding can be seen downstream of the external flow field. The greater the inlet pressure of the internal nozzle, the more intense the entrainment of the external low-speed jet by the internal high-speed jet, and cavitation will also occur at the position of the cylindrical section of the external conical–cylindrical combination nozzle outlet, further promoting the generation of cavitation bubbles. This corresponds with the numerical simulation findings of Liu et al. [45], who found that the external nozzle outlet of an artificially submerged nozzle also produces a cavitation effect. The annular jet of the external nozzle can efficiently enlarge the low-pressure region afterward to the outlet of the internal nozzle, effectively expanding the outlet wall of the cavitation nozzle and enhancing the shear effect between the jets. This develops a low-pressure vortex ring upon the jet’s interaction with the atmosphere, hence enhancing the cavitation effect.

### 4.4. Influence of Input Pressure of Internal Nozzle on Axial Velocity

**Figure 7:**
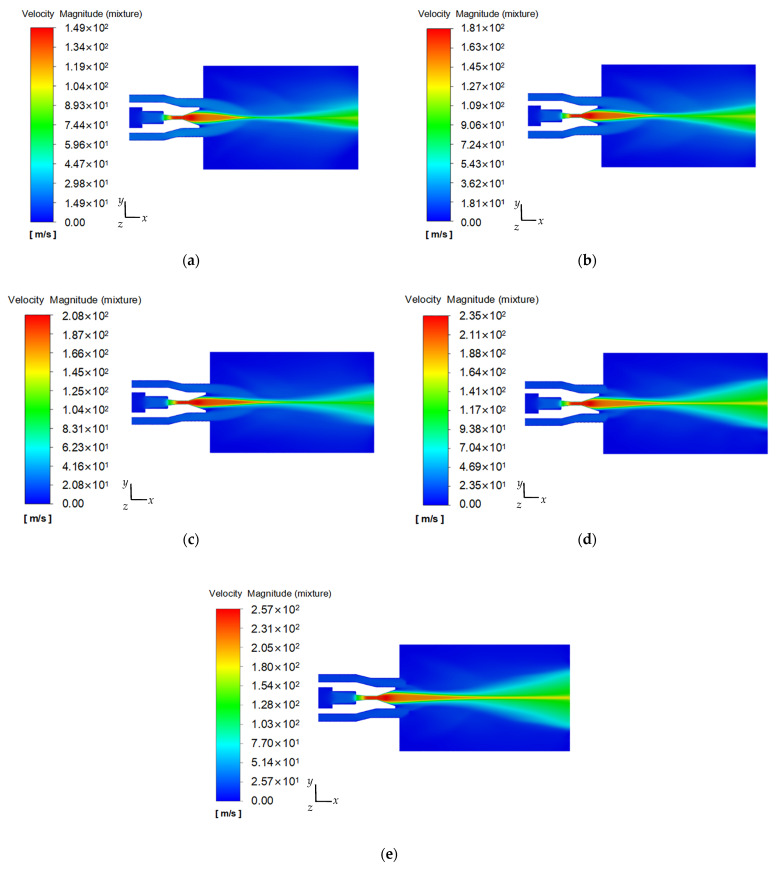
Cloud diagram of axial velocity distribution at different inlet pressures of (**a**) 10 MPa, (**b**) 15 MPa, (**c**) 20 MPa, (**d**) 25 MPa, and (**e**) 30 MPa.

Figure 7 gives a cloud diagram of the axial velocity distribution in the flow farm for the co-current cavitation water jet nozzle with the external nozzle input pressure of 3 MPa and the internal nozzle input pressures of 10, 15, 20, 25, and 30 MPa, respectively.

Figure 7 illustrates that the axial velocity distribution for the flow field in the homemade co-current cavitation water jet nozzle exhibits similarity across varying input pressures, the axial velocity distribution cloud map is symmetrical, and the maximum axial velocity of the flow field gradually rises with the augmentation of the inlet pressure of the internal nozzle. Specifically, the low-pressure peripheral flow first accelerates through the conical convergent section of the external conical cylindrical combination nozzle structure. Then, it intersects with the high-pressure cavitating jet at the position of the diffusion section at the outlet of the internal organ pipe self-oscillation angular cavitation nozzle. The low-pressure peripheral flow, on the one hand, plays a role in slowing down the high-pressure cavitation jet into the atmospheric environment of the “package” role; on the other hand, it plays a role in the high-pressure cavitation jet to continue to provide a low-velocity environment of the body of water so as to ensure the cavitation phenomenon produced by the “shear” effect, so that the main body of the jet flow rate by the external conical–cylindrical combination of the nozzle outlet into the atmosphere can maintain a certain distance. As the jet continues to move toward the atmosphere, the internal and external flow beams of the volume of suction and doping phenomenon intensified and the peripheral low-pressure flow beams showed a gradual contraction trend. The isocore velocity of the jet downstream appears to be a “necked” phenomenon, which is due to the vortex structure formed by the significant shear effect between the fluids, making the flow field appear to be a velocity component parallel to the *y*–*z* plane. Due to the atmospheric environment’s continuous action, the jet core’s velocity decays and the pairing and merging process between the vortices intensifies, causing the shear stress between the fluids to weaken gradually and the momentum exchange within the shear layer to become more violent. When the external nozzle input pressure is 3 MPa and the internal nozzle input pressures are 10, 15, 20, 25, and 30 MPa, the maximum axial velocities in the nozzle flow field are 148, 181, 208, 235, and 257 m/s, respectively.

Subsequently, as illustrated in Figure 7, when the internal jet traverses the exit cylindrical section of the internal cavitation nozzle, the jet axial velocity reaches the maximum; when the jet continues to move to the exit diffusion section of the internal cavitation nozzle, due to the shear effect caused by the velocity gradient between the inner and outer flow beams, it results in significant fluctuations in the jet velocity between the interior and exterior flow beams, and the axial velocity begins to decrease. This is consistent with Luo et al. [22], who found that the axial velocity of the flow field of an artificially submerged cavitation jet nozzle shows a significant stratification phenomenon due to the shear effect, and the axial velocity of the flow field is the highest along the position of the jet axis and progressively diminishes along the axis to the two sides.

### 4.5. Effect of Inlet Pressure of Internal Nozzle on Radial Velocity

**Figure 8:**
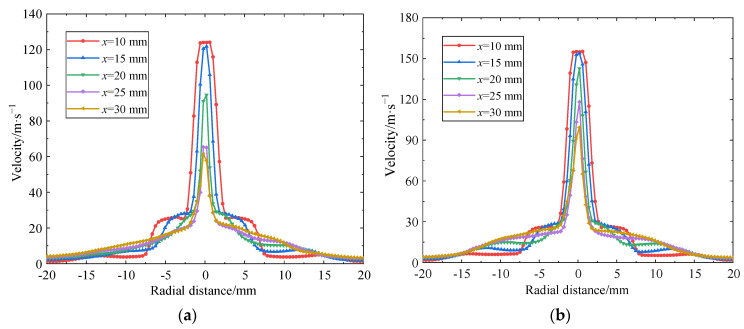

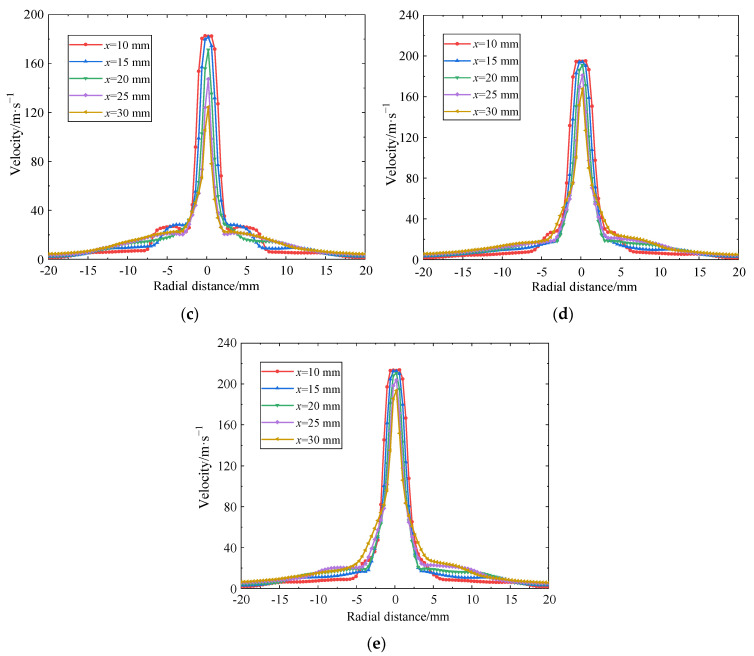
Radial distribution of jet velocity at different inlet pressures of (**a**) 10 MPa, (**b**) 15 MPa, (**c**) 20 MPa, (**d**) 25 MPa, and (**e**) 30 MPa.

Figure 8 shows the radial velocity distribution curves of five sections with *x* = 10, 15, 20, 25, and 30 mm away from the outlet of the internal cavitation nozzle along the jet direction when the inlet pressure of the external nozzle is 3 MPa and the inlet pressure of the internal nozzle is 10, 15, 20, 25, and 30 MPa, respectively.

As shown in Figure 8, the radial velocity distribution curves of flow fields at different sections exhibit good symmetry, and the jet velocity gradually decreases along the radial direction. At section *x* = 10 mm, which is closest to the nozzle outlet, the section is in the initial stage of the jet, exhibiting good convergence performance. The jet has a large velocity gradient in this section, and the shear effect between fluids is very strong, which is conducive to the formation of vortex structures and provides a prerequisite for the occurrence of cavitation phenomena. When the jet moves to the *x* = 30 mm section, the velocity gradient in the section is significantly reduced, and the shear effect is significantly weakened, indicating that the effect of artificial submerged cavitation is negatively correlated with the target distance.

Figure 8 also illustrates that when the input pressure of the internal organ tube self-oscillating angular cavitation nozzle increases, the maximum radial velocity of each radial section is significantly increased and the velocity gradient at the remote section (*x* = 25, 30 mm) is significantly increased, which is conducive to enhancing the shear effect between the inner and outer flow beams. As the input pressure of the inner nozzle rises, the core velocity of the jet increases from 140 m/s to 240 m/s, which intensifies the mixing and shear effect of the inner and outer fluids and is more conducive to the formation of vortex structures under negative pressure. The internal high-speed jet near the axis will move outward in the radial direction, and the external low-speed jet will move inward in the radial direction, thus forming a state in which the external low-speed jet wraps the internal high-speed jet, delaying the divergence of the jet and increasing the energy and length of the jet beam, which is conducive to maintaining the cavitation effect and erosion performance of the cavitating jet in the atmospheric environment. This aligns with the conclusion of Liu et al. [46] that the low-speed water flow around the periphery wraps the high-speed jet inside at the initial stage of the artificially submerged nozzle jet, which can delay the jet divergence and increase the length of the jet beam.

### 4.6. Visualization Test to Verify Cavitation Jet

**Figure 9:**
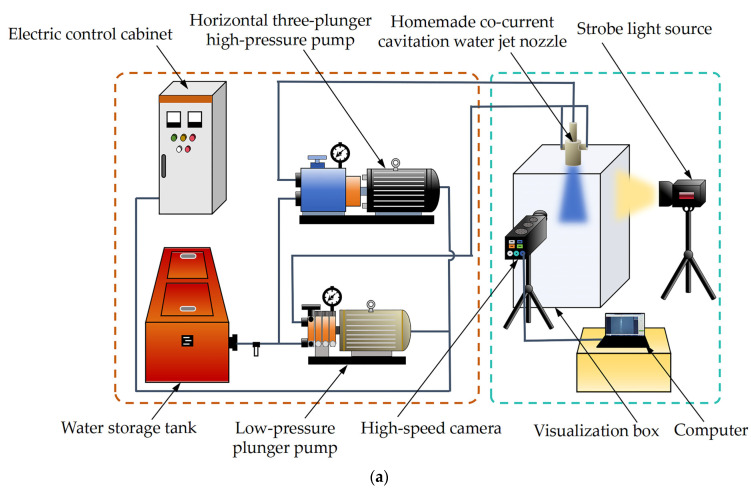

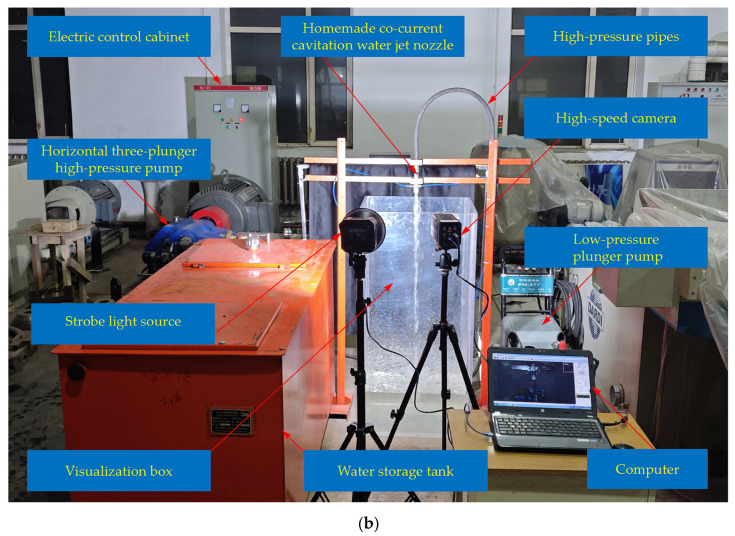
Visualization test system diagram of co-current cavitation water jet nozzle. (**a**) Visualization of system diagram. (**b**) Visualization of system physical photos.

Figure 9a illustrates the visualization test system of the homemade co-current cavitation water jet nozzle. As can be seen from Figure 9a, the system primarily comprises a water storage tank, a low-pressure plunger pump, a horizontal three-plunger high-pressure pump, high-pressure pipes, a homemade co-current cavitation water jet nozzle, a high-speed camera, a strobe light source, a computer, and a visualization box. During the experiment, the external conical–cylindrical combination nozzle of the homemade co-current cavitation water jet nozzle had an inlet pressure of 3.0 MPa, and the internal organ pipe self-oscillating angular cavitation nozzle inlet pressure was selected to be 25 MPa. To fully capture the cavitation cloud‘s dynamic development, the camera was positioned directly opposite the water jet during filming, and a black background cloth was placed on the visual box wall opposite the camera to improve light absorption. The frame rate of the high-speed camera was set to 4000 fps, the shooting interval was 0.25 ms, and the exposure time was 198 μs. Dimensional calibration was performed to facilitate subsequent quantitative analysis of the shooting results. The high-velocity camera recorded real-time photos of the shape of the high-speed gas–liquid two-phase fluid cavitation cloud injected into the visualization box by a homemade co-current cavitation water jet nozzle. A physical photograph of the system is shown in Figure 9b.

The jet generated by the homemade co-current cavitation water jet nozzle is a free jet discharged into the air, distinguishing it from the traditional submerged cavitation jet introduced into stagnant water. The inner and outer jets of the co-current cavitation water jet are on the same axis and have significant differences in speed and pressure. This causes the inner and outer jets to shear against each other and exchange much energy. Disturbed by external flow beams, conventional image analysis and processing techniques for visualizing cavitation clouds, such as the binary image method and the frame difference method [47], cannot clearly and accurately analyze images of cavitation clouds. Therefore, for the detection of cavitation under non-submerged conditions, researchers have proposed the pseudo-color image processing technique, which has been applied to the cavitation detection of centrifugal pumps and has become a common means of fault diagnosis for centrifugal pumps [48]. Pseudo-color image processing is an image processing technique that assigns specific colors to different gray value regions through specific mathematical relationships, thereby converting grayscale images into color images. The purpose is to improve the discernment of image details and enhance the visualization effect of the image through the color expression of the data. This study used the pseudo-color image processing technique to not only visually reveal the macroscopic structural characteristics of the non-submerged cavitating jet but also to capture the subtle dynamic changes in the non-submerged cavitating cloud. The specific processing method is a spatial domain gray-color transformation method based on a pseudo-color image processing technique. First, the original image is processed in grayscale to obtain a grayscale map. Then, the grayscale map is subdivided into multiple subregions. Each subregion is independently transformed in the red, green, and blue (RGB) color transformations, with the three-color transformation processes being independent of each other, allowing for the fine control of each color, thereby achieving a precise enhancement of image details and obtaining the dynamic change law of the cavitation cloud of a homemade co-current cavitation water jet nozzle in non-submerged conditions.

**Figure 10:**
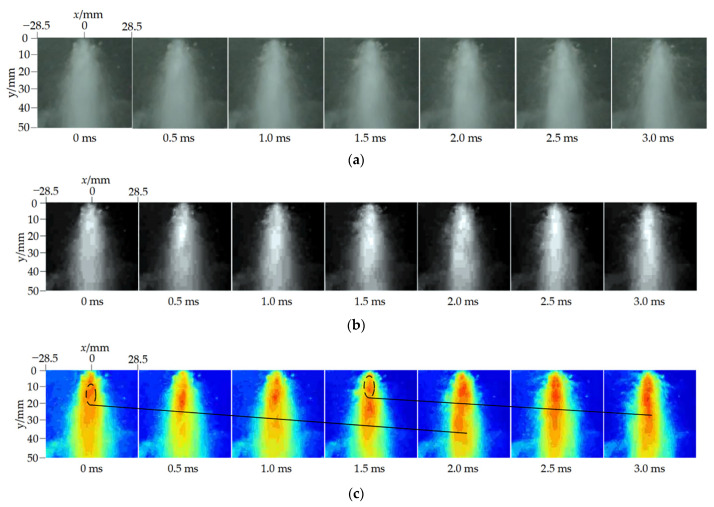
Dynamic evolution process diagram of cavitation cloud. (**a**) Original images. (**b**) Grayscale images. (**c**) Pseudo-color images.

Figure 10 shows the original images, grayscale images, and pseudo-color images of the cavitation cloud‘s dynamic development in the co-current cavitation water jet during two consecutive periods, with the external nozzle inlet pressure at 3.0 MPa and the internal nozzle inlet pressure at 25 MPa. As seen in the original images of Figure 10a, the overall shape of the external flow of the co-current cavitation water jet is conical and divergent. The presence of the external flow has a better binding effect on the internal jet. The jet continues to move downstream, maintaining a certain width against the black background. The grayscale images in Figure 10b illustrate that the external low-speed flow of the co-current cavitation water jet encases the internal high-speed jet emanating from the nozzle exit, subsequently dispersing into the atmosphere. A distinct velocity gradient exists between the internal and external flow beams, exacerbating the turbulent shear of the fluid and facilitating the progression of cavitation. The grayscale image presents the luminous irregular white cavitation cloud on a black background, characterized by extensive coverage and density. The pseudo-color images in Figure 10c demonstrate that the black line segments, extending from left to right, delineate the cavitation clouds during the first and second consecutive periods, encapsulating the entire cavitation process from birth to growth, shedding, and collapse within the wireframe. Specifically, at *t* = 0 ms, the primary cavitation bubbles converge at the outlet of the homemade co-current cavitation water jet nozzle. Since the vortex structure has yet to develop in this area, the cavitation bubble size is relatively small. As the shear force increases, the cavitation bubble enters the development stage and the size and density of the cavitation cloud gradually increase. The cavitation cloud further expands to its maximum width at *t* = 0.5–1.5 ms. As the turbulence intensifies between the inner and outer flow beams, the combined action of turbulence, coherent vortices, and atmospheric drag affects the cavitation cloud. The end of the cavitation cloud becomes accumulated and asymmetric, eventually leading to a gradual decrease in the size of the cavitation bubble and the shedding and collapse of the cavitation cloud. At the same time, a new upstream cavitation cloud cluster is born and begins to develop. At *t* = 2 ms, the cavitation cloud of the previous period almost wholly disappears and the cavitation cloud of the next period develops rapidly. The cavitation cloud of the next period will continue the development process of birth–growth–shedding–collapse of the previous period, which is similar to the evolution law of the cavitation cloud obtained by Cui et al. [49] in a visual experiment of the cavitation water jet. The visualization test results indicate that the cavitation bubble development process of the non-submerged cavitation jet generated by the homemade co-current cavitation water jet nozzle exhibits notable continuity, periodicity, and regularity, resulting in an effective cavitation effect and confirming the validity of the numerical simulation results.

## 5. Conclusions

This study designed a homemade co-current cavitation water jet nozzle structure, which consists of an internal organ pipe self-oscillating angular cavitation nozzle and an external conical–cylindrical combined nozzle, to enhance the cavitation ability of the cavitating jet in the atmospheric environment (non-submerged condition). The gas–liquid two-phase flow field of the nozzle was solved using the CFD method, and it was compared with the existing artificial submerged cavitating nozzle to verify the rationality of the homemade co-current cavitation water jet nozzle design.

The volume fraction of vapor generated by the co-current cavitation water jet nozzle is distributed axially symmetrically, which has a positive correlation with the internal nozzle’s inlet pressure. When the external nozzle input pressure is 3 MPa and the internal nozzle input pressures are 10, 15, 20, 25, and 30 MPa, the vapor volume percentages generated by the nozzle are 95.2%, 95.6%, 95.7%, 95.9%, and 96.4%, respectively, which shows that the homemade co-current cavitation water jet nozzle has a good cavitation performance.

The pseudo-color images of the cavitation cloud, generated by the homemade co-current cavitating water jet nozzle, reveal a significant periodic dynamic change rule following the application of the spatial domain gray-color transformation method. One change period is 1.5 ms, which indicates that the nozzle can generate a stable cavitating jet in a non-submerged condition, verifying the accuracy of the CFD numerical simulation results.

## Figures and Tables

**Table 1:** Flow field mesh irrelevance test results.

Number of Meshes/Pcs	Maximum Vapor Volume Percentage/%
1,049,513	92.0
1,299,513	94.6
1,549,513	95.2
1,799,513	95.2
2,049,513	95.1

## Data Availability

The data presented in this study are available upon request from the corresponding author. As the data in this paper belong to the National Natural Science Foundation of China, it involves related privacy and is not owned by individuals.
